# Recycling the recyclers: lysophagy emerges as a new pharmacological target for retinal degeneration

**DOI:** 10.1080/15548627.2024.2391726

**Published:** 2024-08-12

**Authors:** Juan Ignacio Jiménez-Loygorri, Patricia Boya

**Affiliations:** a Department of Cellular and Molecular Biology, Centro de Investigaciones Biológicas Margarita Salas, CSIC, Madrid, Spain; b Department of Neuroscience and Movement Science, Faculty of Science and Medicine, University of Fribourg, Fribourg, Switzerland

**Keywords:** AMD, autophagy, lysosomal membrane permeabilization, lysosome, RPE

## Abstract

Dysregulated macroautophagy/autophagy is one of the hallmarks of aging and has also been linked to higher incidence of several age-associated diseases such as age-related macular degeneration (AMD). The main cell type affected in AMD is the retinal pigment epithelium (RPE), and this disease can lead to central vision loss. Despite affecting around 8.7% of the population between 45–85 years, its etiopathogenesis remains unknown. In our recent manuscript using the pharmacological sodium iodate (SI) model of AMD we identified severe lysosomal membrane permeabilization (LMP) in the RPE, that leads to autophagy flux blockage and proteostasis defects. Treatment with the natural compound urolithin A (UA) reduces RPE cell death and alleviates vision loss, concurrent with full autophagy restoration. While UA was initially described as a specific mitophagy inducer, we now show that it is also able to promote SQSTM1/p62-dependent lysophagy in the context of lysosomal damage and LMP. Genetic downregulation of SQSTM1/p62 fully abolishes the effect of UA on lysophagy while mitophagy stimulation remains unaffected. In summary, these findings highlight the wide range of pathways modulated by UA and its potential implementation in the management of AMD and other diseases involving lysosomal damage.

Age-related macular degeneration (AMD) is a retinal disease characterized by progressive bilateral degeneration of the macula, a region of the central retina in charge of high detail, colored vision. Its progression has been associated with genetic factors (e.g., *CFH*^*Y402H*^ and *ARMS2*^*A69S*^ variants) and environmental factors (e.g., smoking, diet). AMD affects around 200 million patients worldwide, and its incidence is expected to double by 2040 due to population aging. Within the outer retina, a thin polarized monolayer of epithelial cells termed retinal pigment epithelium (RPE) undergoes rapid degeneration during the early stages of AMD but the cause of this selective vulnerability remains obscure. The RPE provides trophic support to neighboring photoreceptors and also undertakes the daily recycling of visual pigments, and its malfunction eventually leads to secondary photoreceptor cell death and vision loss, a phenomenon called geographic atrophy. Mitochondrial dysfunction and impaired autophagy have been observed in primary RPE cultures from donors with AMD.

Previous work from our lab has described the natural polyphenol urolithin A (UA) to be neuroprotective in the context of physiological aging in the retina, helping preserve visual function and alleviating neuroinflammation in a mitophagy-dependent manner. Encouraged by these findings, we tested the neuroprotective potential of UA in the sodium iodate (SI) model of geographic atrophy [1]. Indeed, UA promotes RPE survival *in vivo* in C57BL/6J mice and *in vitro* in the human ARPE-19 cell line. Mice co-treated with UA show decreased apoptotic photoreceptor cell death, increased cone survival and, ultimately, preserved day (photopic) and night (scotopic, mixed) vision when compared to the vehicle group that only received SI. *mito*-QC reporter (mCherry-GFP-FIS1[101-152]) analysis revealed that both SI damage and UA elicit PINK1-PRKN-dependent mitophagy, and we also determined that UA is able to simultaneously boost mitochondrial biogenesis and alleviate ΔΨm alterations. However, we observed that mitochondria enriched with PINK1 and PRKN accumulate over time in SI-treated cells and, surprisingly, knockdown (KD) of either *PRKN/PARK2* or *PINK1* do not abolish the pro-survival effect of UA. These data indicate that the neuroprotective mechanism of action of UA in SI-induced geographic atrophy is mitophagy-independent.

The accumulation of PINK1-positive mitochondria is suggestive of disturbed delivery of autophagic cargo to the lysosome. To address this, we analyzed general macroautophagy using the reporter mCherry-GFP-MAP1LC3B and identified a very severe accumulation of autophagosomes in SI-treated cells and mice, that is fully reverted when UA is added. Lysosomal degradation is the limiting step of autophagy and, while there are no changes on overall lysosomal mass, we observed a decrease in the number of acidic lysosomes and, conversely, a dramatic increase in the number of permeabilized LGALS3 (galectin 3)-positive lysosomes. Notably, both of these observations are again reverted upon UA treatment. Cells have different response mechanisms to cope with lysosomal membrane permeabilization (LMP) depending on damage severity and ranging from ESCRT-mediated membrane repair to lysosomal biogenesis or lysophagy (Figure 1), the selective autophagic degradation of permeabilized lysosomes within healthy lysosomes. We discarded ESCRT-mediated membrane repair due to the very acute and widespread LMP observed. Treatment with SI does induce TFEB nuclear translocation and activation of the CLEAR network transcriptional response, suggesting that cells boost lysosomal biogenesis to bypass the LMP, but this is not the case when cells are co-treated with SI and UA or UA alone.

**Figure 1.**
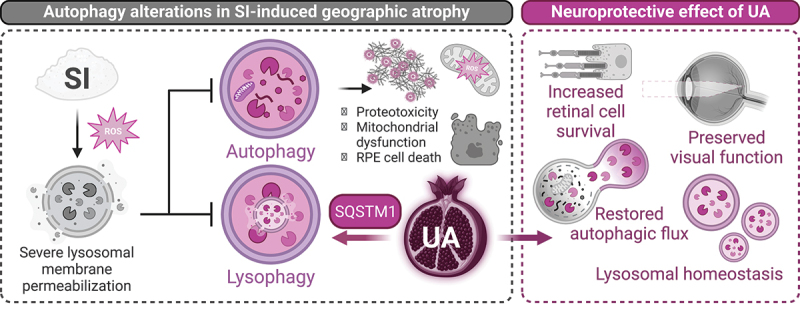
Lysophagy mediates the neuroprotective effect of urolithin a (UA) in the sodium iodate (SI) model of AMD-associated geographic atrophy in an SQSTM1/p62-dependent manner. SI induces severe lysosomal membrane permeabilization *in vivo* and *in vitro* causing a disruption of autophagic flux and RPE cell death. Treatment with UA stimulates SQSTM1/p62-dependent lysophagy, abrogates autophagy defects and promotes retinal cell survival (RPE, photoreceptors) leading to preserved visual function. Diagram created with BioRender.com.

Using the lysophagy *tfGal3* reporter (GFP-RFP-LGALS3), that makes it possible to differentiate between LMP-positive lysosomes and healthy lysosomes taking up permeabilized lysosomes, we determined that SI fully blocks lysophagy in RPE cells and UA is able to restore lysophagic flux. Genetic manipulations helped us to identify the ubiquitin receptor SQSTM1/p62 as the essential pro-survival effector of UA, while another previously described lysophagy mediator (*UBE2QL1*) is dispensable. SQSTM1/p62 had previously been described as the main ubiquitin receptor involved in lysophagy and has recently been shown to undergo phase separation at the lysosomal membrane, generating a platform that favors the recruitment of autophagy machinery. KD of SQSTM1/p62 leads to exacerbated LMP in SI-treated cells and fully abrogates lysophagy flux in control and UA-treated cells. SQSTM1/p62-KD has no effect on general autophagy or PINK1-PRKN-dependent mitophagy induced by UA.

In conclusion, these results highlight the multiple selective autophagy pathways activated simultaneously by UA and their cell type-, context-dependent relevance. The mechanism by which UA stimulates SQSTM1/p62-dependent lysophagy remains to be elucidated. While we did not observe a transcriptional upregulation, there is an increase in SQSTM1/p62-positive foci formation suggesting that UA may promote its condensation. Most importantly, UA-induced lysophagy restores autophagic flux, decreases RPE cell death and helps preserve visual function during AMD-associated geographic atrophy. Lysophagy stimulation with UA warrants further exploration in other diseases characterized by lysosomal dysfunction such as *retinitis pigmentosa*, Alzheimer disease, Parkinson disease or sarcopenia.
